# Intrascleral Suture Anchoring: A Flapless/Grooveless Four-Point Intraocular Lens Fixation Technique

**DOI:** 10.1155/2020/6642007

**Published:** 2020-12-01

**Authors:** Xin Hu, Bo Zhao, Haiying Jin

**Affiliations:** ^1^Department of Ophthalmology, Huaihe Hospital, Henan University, Kaifeng 475000, China; ^2^Department of Ophthalmology, Shanghai Tenth People's Hospital Affiliated to Shanghai Tongji University School of Medicine, Shanghai 200072, China

## Abstract

**Purpose:**

We describe a minimally invasive suture fixation technique for four-point fixation of intraocular lenses (IOLs) in the treatment of aphakic eyes, namely, the intrascleral suture anchoring technique. Neither scleral flaps nor large conjunctival dissections are required.

**Methods:**

This study included 11 eyes (11 patients). After looping the eyelets on the IOL haptics and externalizing the threads, the curved needle attached to the externalized thread was started with two sequential intrascleral passes from the first fixation point to reach the second fixation point. The same procedure was performed for the other side of the IOL. A fixation knot was created in the sclerotomy by the two ends of the thread to close the suture loop for IOL fixation. Another knot was created about 2 to 3 mm from the exiting point and was intrasclerally anchored by the aid of the attached curved needle.

**Results:**

The mean postoperative follow-up period was 9.7 ± 5.8 months (range 5–15 months). The IOLs of all eyes remained well positioned and stable postoperatively. The postoperative visual acuities were improved. No suture erosion, suture loosening, hypotony, scleral atrophy, chronic inflammation, retinal tear, and/or detachment were observed within the follow-up period.

**Conclusion:**

The present technique is an alternative, flapless method for the four-point suture fixation of IOLs. It provides both minimal surgical trauma and reliable stability.

## 1. Introduction

Intraocular lens (IOL) implantation in cases of insufficient capsular support after lens extraction is challenging. Various techniques are used to fixate the IOL within the eye, including transscleral suture fixation, [1–3] intrascleral fixation of IOL haptics, [4] flanged fixation, [5] glued IOL technique, [6] iris suture fixation of posterior chamber IOLs [7], and iris claw fixation [8]. Each method has its advantages and disadvantages. The four-point IOL fixation using two suture loops to fixate the four haptics has been reported to provide a variety of advantages, including enhanced IOL stability and centration, avoidance of IOL tilt and postoperative pupil capture, and low risk of cystoid macular edema and pigmentary dispersion glaucoma [9–13]. As there are four fixation points at the sclera, excessive conjunctival dissections and large scleral flaps are commonly performed to cover the exterior threads and knots of the fixating suture loops. These dissections and scleral manipulations are surgically traumatic. In cases with previously performed surgeries or ocular trauma, scarring of the conjunctiva-scleral tissue may add to the difficulty of performing conjunctival dissections and scleral flaps. An alternative approach to avoid creations of large scleral flaps is to leave the exterior threads on the surface of sclera and bury the knot by rotating it into the sclerotomy [11, 12]. The exterior threads are covered by conjunctiva; however, there are concerns of suture erosion and postoperative endophthalmitis. Moreover, for cases with excessive conjunctival scarring, the technique is impractical. We present a technique that anchors the sutures intrasclerally. The procedures are performed under the four transconjunctival puncture sites created by using a 30-gauge needle. Using this technique, neither large conjunctival dissections nor manipulations (flaps, grooves, or pockets) on the sclera are required.

## 2. Methods

A retrospective analysis of patients who underwent flapless four-point fixation of dislocated IOLs (Akreos AO60, Bausch and Lomb, North Clearwater, FL) after lens extraction, enrolled between January 2019 and July 2020, was performed. The present study adhered to the tenets of the Declaration of Helsinki and was approved by institutional ethics board of Tenth People's Hospital affiliated to Shanghai Tongji University School of Medicine. Written informed consent was obtained from all patients. All patients provided informed consent after a description of the nature and consequences of the study. Data collection included demographic details, indication for surgery, intraoperative and postoperative complications, follow-up duration, preoperative and postoperative intraocular pressure, visual acuity, IOL position evaluated by anterior segmental photograph and Scheimpflug imaging system (Pentacam, Oculus Optikgeräte GmbH, Wetzlar, Germany), posterior segment photograph, and optical coherence tomography for macular evaluation.

### 2.1. Surgical Technique

**Figure 1:**
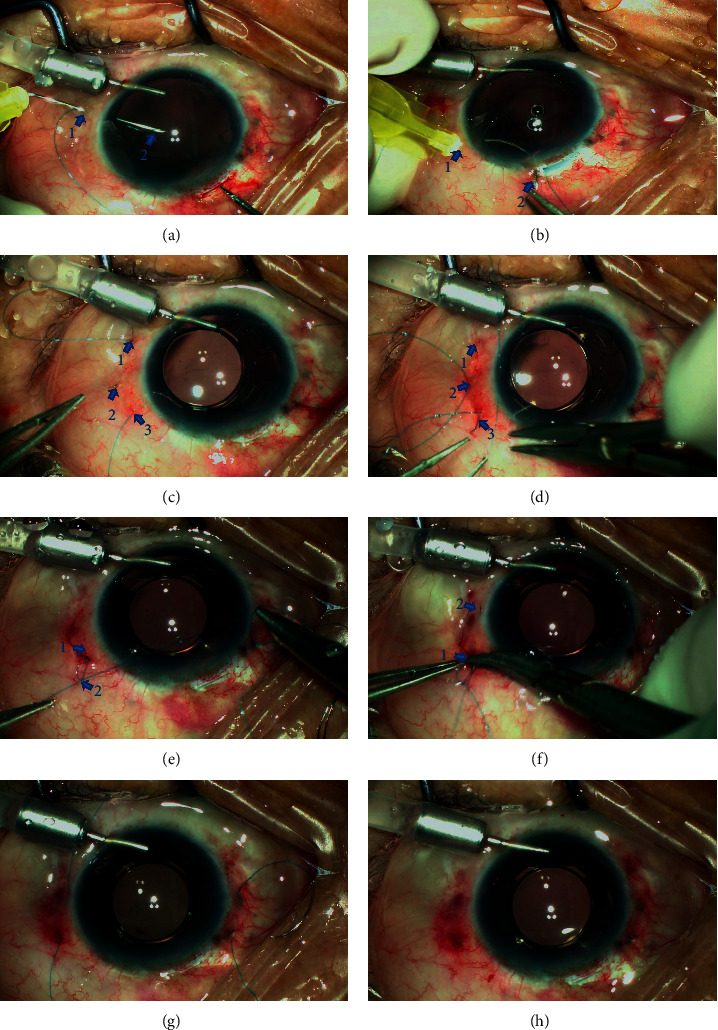
Intraoperative view of the surgical procedures. (a) Introduce the 8–0 polypropylene thread into the eye by the suture-in-needle technique using direct ab externo puncturing of a 30-gauge needle. Arrow 1: the first fixation point; arrow 2: the loop of the suture can be grasped by using forceps to retrieve it from the main incision. (b) After looping through the eyelets of the IOL, the thread is externalized by the curved-needle-retrieving technique. Arrow 1: the second fixation point; arrow 2: the tip of the curved 30-gauge needle. (c) After externalizing the four fixation sutures and implanting the IOL into the posterior chamber, the curved needle attached to the suture is held by a needle holder and intrascleral pass from the sclerotomy of the first fixation point (arrow 1) to the adjacent sclera (arrow 2) is performed. Arrow 3: the second fixation point. (d) Perform a relay intrascleral pass from the exit point (arrow 2) to exit the needle from the second sclerotomy (arrow 3). (e) Create the overhand fixation knot (arrow 1) by the two ends of the sutures to fixate the IOL. Create a second knot (arrow 2) 3 mm from the exit point and perform the intrascleral incarceration of the knot. (f) Perform the intrascleral pass of the curved needle attached to thread and knots from the second fixation point (arrow 1) to the adjacent sclera (arrow 2). (g) After pulling the thread to lead the threads and knots into the scleral tunnel, only four threads remain from the sclera. (h) After cutting all the externalized threads flush to the sclera, the IOL is well centered. Conjunctival incisions are left sutureless.

Surgeries were performed under general anesthesia (2 cases) or retrobulbar anesthesia (9 cases) by one of us (J.H.).  Figure 1 and the supplemental video demonstrate the procedures (see Supplementary Materials (Available here)). An 8–0 polypropylene thread (Prolene, Polypropylene Suture; Ethicon, Johnson-Johnson, New Brunswick, NJ) with a curved needle was bisected in its middle. The procedures of introducing the thread into the eye and looping the eyelets on the haptics of the Akreos AO60 (Bausch and Lomb, North Clearwater, FL) foldable posterior chamber IOL were similar to those of previously published methods [14, 15]. The suture-in-needle technique was performed to introduce the thread into the eye. The free end of the 8–0 polypropylene thread was threaded approximately 3-4 mm into the tip of a 30-gauge needle. A direct transconjunctival ab externo puncture of the 30-gauge needle was performed at the first fixation site to introduce the loop of the suture into the eye, leaving the other end of the thread connected to the curved needle exterior to the eye. The loop of the suture was then grasped by forceps introduced through the main incision. The end of the suture in the 30-gauge needle was taken out from the main incision using forceps. The 30-gauge needle was withdrawn from the eye. The suture was then looped though eyelets of both haptics of the IOL on the left side. Another 30-gauge needle was curved by using a needle holder. An ab externo transconjunctival puncture of the curved 30-gauge needle was performed at the second fixation point, and then its tip was guided out from the main incision by using forceps. The curved 30-gauge needle avoids the distortion of the globe during pass-through of the sclera and the main incision that occurs when using a straight needle. The end of the polypropylene thread (after looping the eyelets of the IOL) was inserted into the lumen of the curved 30-gauge needle from its tip. After withdrawing the needle, the end of the suture was externalized from the second fixation point. The same procedures were repeated to loop the haptics and externalize the ends of the thread on the other side of the eye. After folding and implanting the IOL into the posterior chamber, the intrascleral suture anchor technique was performed. The conjunctival incisions of the second and fourth fixation points were slightly enlarged by blunt dissection to expose the underlying sclera. The curved needle attached to the exterior suture was held by a needle holder and was then started with an intrascleral pass from the first fixation site of the sclerotomy to the adjacent transscleral penetration site parallel to the limbus. The tip of the needle was then pulled out transconjunctivally using a needle holder. A second intrascleral pass of the needle from the exiting point of the sclera to the second fixation point was performed in a relaying manner. The tip of the curved needle was then passed out from the sclerotomy of the second fixation point. The two ends of the same suture loop thus converged from the same coincident sclerotomy. The coincident may not be accomplished on the first manipulation of the relaying intrascleral pass; however, by withdrawing the needle tip back into the scleral tunnel and adjusting the length and direction of the needle track, it can be easily accomplished in a second maneuver. The same manipulations were performed for the other side of the eye. Thus, the two suture loops were formed to fixate the IOL for both sides. After adjusting the tensions of the suture loops to center the IOL, a 2-1-1 overhand fixation knot was created in the sclerotomy by the two ends of the thread to close the suture loop for each side. Another overhand knot (friction knot) was then created about 2 to 3 mm from the first fixation knot. The technique of anchoring the friction knot into the scleral tunnel to bury the ends of the thread was identical to our previous publication [16]. A 27-gauge needle with a sharp beveled tip was used to create a wage-shaped intrascleral tunnel from the sclerotomy approximately 3-4 mm in length parallel to the limbus. Avoid accidently cutting the sutures by staggering the 27-gauge scleral tunnel from the previous needle track of the buried sutures. The curved needle connected to the overhand knot was then held by a needle holder and was started with an intrascleral pass from the sclerotomy to the adjacent transscleral penetration site through the scleral tunnel. The needle was then pulled out transconjunctivally. The thread was then further pulled to lead the friction knot tucked into the scleral tunnel. The same manipulations were performed for the other side. After cutting the four externalized ends of the threads flush to the scleral surface, all the exterior threads and knots were anchored in the intrascleral needle tracks. The small conjunctival openings were left sutureless.

## 3. Results

**Figure 2:**
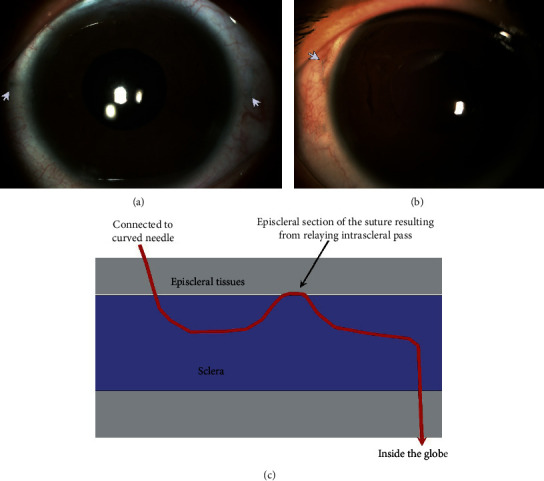
Postoperative overviews. There was barely conjunctival and scleral scarring after surgery. Only very short sections of the thread (arrows) resulting from the relaying intrascleral pass can be observed under the episcleral tissue in some of the cases. This phenomenon is identical to the turning point of the *Z*-suture technique.

A total of 11 eyes of 11 patients (6 men and 5 women) were included. The mean age was 36.0 years (±standard deviation, 21.7; range, 10–68 years). The mean follow-up period was 9.7 ± 5.8 months (range 5–15 months). The indications for surgery included aphakia after pars plana vitrectomy (PPV) and retinal repair secondary to traumatic retinal detachment (*n* = 3), aphakia after PPV secondary to traumatic dislocated crystalline lens and vitreous hemorrhage (*n* = 2), dislocated crystalline lens due to Marfan syndrome (*n* = 2), aphakia after PPV secondary to traumatic dislocated crystalline lens (*n* = 1), and aphakia after PPV and repair of cyclodialysis secondary to traumatic dislocated crystalline lens, vitreous hemorrhage, and cyclodialysis (*n* = 1). The preoperative logarithm of the minimum angle of resolution visual acuity was 1.12 ± 0.47 (Snellen 20/264). The logarithm of the minimum angle of resolution visual acuity at the final follow-up was 0.48 ± 0.24 (Snellen 20/60). No intraoperative complications were observed except a transient mild ciliary hemorrhage during the needle puncture in one eye. No evidence of suture erosion, suture loosening, hypotony, scleral atrophy, chronic inflammation, or retinal tear and/or detachment was observed in any of the patients. The IOLs were well centered within the follow-up period. The sutures were invisible in most of the cases (*n* = 8) at the final examination. Very short sections (about 0.5 mm) of the sutures resulting from the relaying intrascleral pass were observed (*n* = 3) with careful examination under the episcleral tissues; however, these sections were barely visible (Figure 2).

## 4. Discussion

Various innovations in IOL suture fixation in patients with insufficient capsular support have reduced surgical trauma and improved long-term postoperative stabilization; however, the technique still has limitations and is technically challenging. The use of 8–0 or 9–0 polypropylene thread or polytetrafluoroethylene thread reduces the risk of postoperative IOL redislocation resulting from suture breakage of the 10–0 polypropylene thread. Surgical trauma of the scleral suture fixation is reduced by two flapless techniques: the *Z*-suture and the friction knot techniques [17, 18]. The four-point fixation of IOL with four-eyeleted haptics provides high stability and centration and avoids tilt and pupil capture of the IOL, thereby avoiding the limitations of the conventional two-point fixation. However, as there are four fixation points on the sclera, the technique commonly involves large conjunctival dissections and scleral flaps to cover the exterior threads and knots of the two suture loops, which results in excessive surgical trauma [9–13]. Although a less traumatic technique has been advocated, which involves leaving the exterior threads on the surface of the sclera and rotating the knot into the sclerotomy, it has the risks of suture erosion and endophthalmitis due to the subconjunctival sutures externalizing directly from the sclerotomies [11, 12]. Moreover, for cases with conjunctival scarring or atrophic Tenon's capsule, the technique is impractical.

To inherit the advantages of four-point fixation and to reduce surgical trauma, we present an intrascleral suture anchoring technique. The advantages of this technique are multifold. First, the technique uses 8–0 polypropylene thread [13, 15, 16]. The thread is thicker and has a higher tensile strength than the 10–0 polypropylene thread and thus improves intraoperative manipulations and reduces late IOL dislocation due to suture breakage. Second, the exterior knots and sutures are anchored intrasclerally without exposure. The sutures were invisible in most of the cases at the final examination. Only very short sections of the thread resulting from the relaying intrascleral pass were observed that was covered by episcleral tissue in some of the cases (Figure 2), which is similar to the turning point of the *Z*-suture technique and does not relate to any side effect [17]. Third, the securing of the sutures is accomplished by overhand knots. By adjusting the tensions of the bilateral suture loops before fastening the knots, the centration of the IOL could be acquired. Fourth, unlike the conventional suture technique fixation methods that secure the suture by lamellar scleral tissue beneath the scleral flap, there is no risk of late IOL dislocation due to tissue dehiscence induced by the consistent cutting effect of the suture. The intrascleral incarceration of the cutting ends used in the present technique is a modified technique of the friction knot method first published by Oskala [18]. The technique was further modified by us into an overhand friction knot technique to fixate dislocated IOLs [16]. The modified technique was adopted in this research to lead the ends of the threads anchored into the scleral tunnel, which can be a satisfactory approach to bury the knots and cutting ends of the suture without creating scleral flaps, pockets, or grooves. The manipulations can be performed under the sutureless small conjunctival incisions; therefore, the present technique greatly reduced the surgical trauma noted in other techniques that require the creations of large conjunctival incisions and scleral flaps. As the cutting ends lie tangential to the sclera, the technique inherits the major advantage of the friction knot method of preventing suture erosion. Finally, the present technique presented a method of externalizing the thread using a curved 30-gauge needle, which inherits the advantages of the ab externo technique. The curving modification of the 30-gauge needle avoids distortion of the globe during passing through the scleral fixation point and the main incision (Figure 1(b)). As compared with the technique of retrieving the threads by using 27-gauge or 25-gauge (with or without assistance of trocars) microforceps, the 30-gauge sclerotomies are self-sealing and less traumatic [11, 12]. Therefore, the technique reduces the risk of wound leakage and postoperative hypotony.

In summary, the present technique is an alternative, flapless method for the transscleral four-point fixation of IOLs. It provides both minimal surgical trauma and reliable stability. A study with a longer follow-up time and more cases is required to confirm the long-term stability of this method and compare it with other fixation methods.

## Data Availability

The datasets used and/or analyzed during the current study are available from the corresponding author on reasonable request.

## Supplementary Materials

Supplementary Materials Video Clip 1: intrascleral suture anchoring: a flapless four-point intraocular lens fixation technique.
